# A Public Database of Memory and Naive B-Cell Receptor Sequences

**DOI:** 10.1371/journal.pone.0160853

**Published:** 2016-08-11

**Authors:** William S. DeWitt, Paul Lindau, Thomas M. Snyder, Anna M. Sherwood, Marissa Vignali, Christopher S. Carlson, Philip D. Greenberg, Natalie Duerkopp, Ryan O. Emerson, Harlan S. Robins

**Affiliations:** 1 Adaptive Biotechnologies, Seattle, United States of America; 2 Fred Hutchinson Cancer Research Center, Seattle, United States of America; 3 University of Washington, Seattle, United States of America; Monash University, Australia, AUSTRALIA

## Abstract

The vast diversity of B-cell receptors (BCR) and secreted antibodies enables the recognition of, and response to, a wide range of epitopes, but this diversity has also limited our understanding of humoral immunity. We present a public database of more than 37 million unique BCR sequences from three healthy adult donors that is many fold deeper than any existing resource, together with a set of online tools designed to facilitate the visualization and analysis of the annotated data. We estimate the clonal diversity of the naive and memory B-cell repertoires of healthy individuals, and provide a set of examples that illustrate the utility of the database, including several views of the basic properties of immunoglobulin heavy chain sequences, such as rearrangement length, subunit usage, and somatic hypermutation positions and dynamics.

## Introduction

The diverse B-cell repertoire of a healthy individual allows the recognition of a wide range of antigenic epitopes, resulting in a robust adaptive humoral immune response against pathogens. The vast majority of B lymphocytes express a single unique B-cell antigen receptor (BCR), a heterodimeric protein complex composed of a heavy and a light immunoglobulin chain, each of which contains a highly diverse antigen-binding domain. The human immunoglobulin heavy chain (IgH) locus comprises approximately one megabase of chromosome 14, and contains at least 51 functional variable (V) region genes, 25 diversity (D) genes and 6 joining (J) genes that undergo a series of recombination events to assemble a functional heavy chain[1–3]. This recombination process creates a vast array of antigen-binding receptors through the random assortment of different V, D, and J segments (combinatorial diversity), and the insertion of non-templated (N) and palindromic nucleotides (P) at the junctions between V/D and D/J segments (junctional diversity). Productive in-frame VDJ rearrangements result in a functional heavy chain and lead to a permanent alteration of the genomic DNA sequence of a B cell, defining it as a clone. Similarly, the human immunoglobulin light chain к and λ loci occupy approximately one megabase on chromosomes 2 and 22, respectively, and contain 30–40 V and 4–5 J segments that can recombine to generate a light chain that is assembled with the heavy chain to form a functional receptor, jointly determining the specificity of recognition[3].

This initial BCR repertoire created in naive B cells through combinatorial and junctional diversity increases upon antigen encounter through the process of somatic hypermutation (SHM), which is mediated by activation-induced cytidine deaminase (AID)[4]. As a result, single base substitutions and occasional insertions or deletions occur throughout the rearranged BCR genes, generating a BCR with increased affinity for its antigen [5, 6]. Our understanding of SHM is limited by the relatively small number of BCR sequences from antigen-experienced B cells that have been available until recently.

The clonal diversity of the human BCR repertoire has been difficult to estimate. Early studies relied on extrapolation from the relatively small number of sequences obtained through low-throughput methods such as immunoscope or traditional Sanger-based sequencing (reviewed in [7, 8]). In recent years, high-throughput sequencing (HTS) methods have considerably increased the number of unique BCR sequences available to the scientific community. However, most of the sequences generated to date are not readily available in a centralized and curated database—the most widely used resource of immune loci (International ImMunoGeneTics, or IMGT^**®**^) currently contains approximately 50,000 rearranged human IgH sequences[9]. On the other hand, several other large datasets are publicly available: for example, the National Center for Biotechnology Information (NCBI) Sequence Read Database (SRA, http://www.ncbi.nlm.nih.gov/sra)) includes 454 pyrosequencing data from HIV-1 neutralizing antibodies from the Vaccine Research Center (SRP02639) and antibodies generated in response to influenza vaccination from dbGaP (SRP029381), as well as Illumina sequencing data from healthy donor repertoires from BioProject (SRP037774). In addition to this, a number of publications in the last few years have made considerable numbers of BCR sequences available to the scientific community[10–24]. As a consequence of this recent surge in the number of B-cell sequences available, centralized database and complex data processing and visualization tools are needed to analyze, visualize and interpret these large datasets of immune sequences.

Immunosequencing of the TCR and BCR repertoires has greatly improved our understanding of B- and T-cell biology[25], leading to the refinement and modification of B- and T-cell development models[26–29]. In addition, these data have resulted in multiple clinical advances. For example, immunosequencing has resulted in clinical tests for diagnosis and monitoring of minimal residual disease for lymphoid malignancies[23, 30], has guided the discovery of neutralizing antibodies against HIV[31], has been used to dissect the role of T-cells in autoimmuny[32, 33] vaccination[34] and transplant[35, 36], and to better understand the role of infiltrating T lymphocytes in ovarian cancer[37], melanoma[38] and glioblastoma[39].

Here, we present a public resource of more than 37 million unique immunoglobulin heavy chain (IgH) sequences resulting from the digital amplification and sequencing of the most variable region of the IgH gene from 10 million naive and 10 million memory B cells each from three healthy adult donors, using the immunoSEQ platform[18, 27, 40]. In addition, we have created a suite of software tools that facilitates the visualization and analysis of these data. Using many barcoded replicates for each sample, our method approximates single-molecule sequencing of BCRs at high-throughput, thus ensuring a faithful quantitative representation of nearly all clones present in the biological sample. Besides describing the study design, the specifics of the sequencing technology employed, and the resulting data set, we illustrate the use of the web-based tools developed to enable visualization and analysis of these data.

Finally, to further demonstrate the utility of this resource, we explore a few of the many potential biological questions that can be addressed through our data set: (1) we explored and compared the clonal diversity of naive and memory BCR repertoires at an hitherto unprecedented level of sequencing depth; (2) we confirmed V gene family usage patterns in healthy subjects using a bias-free approach; (3) we examined variations in the length of the third Complementarity Determining Region (CDR3) in naive and memory B-cell populations; (4) we analyzed SHM within the steady-state BCR repertoire; and (5) we deconvoluted patterns of SHM substitutions in V genes for naive and memory cells.

## Materials and Methods

### Sample source and B cell isolation procedure

Whole blood samples were collected from three 25–40 year old Caucasian males participating in a study of healthy human volunteers under approval of the Fred Hutchinson Cancer Research Center Institutional Review Board. The donors did not report any infections or vaccinations in the 6 months previous to sample collection. All donors provided written informed consent. All samples were processed less than 2 hours after venipuncture. Peripheral blood mononuclear cells were separated from 400 mL of whole blood by Ficoll (GE Healthcare) gradient density centrifugation at 400g and 22°C. Next, total B cells were enriched from PBMCs using CD19 MicroBeads and the autoMACS Pro Separator (Miltenyi Biotec). B cells were then stained with anti-CD19APC, anti-CD3FITC, anti-CD27PE, anti-IgM-APC750, and anti-IgD-PECy7 (all from BD BioSciences) and sorted using the BD FACS Aria II with FACSDiva v6.1.3 software (BD BioSciences). Naive (CD19^+^, CD27^-^, CD3^-^, IgM^+^, IgD^+^) and memory (CD19^+^, CD27^+^, CD3^-^) B cells were sorted to a purity of 97% or greater. Sort purity was assessed by passing a small sample of each sorted population back through the flow cytometer. We note that this memory B-cell sort contains all CD27^+^ B cells, including both class-switched and IgM memory B cells. Representative flow cytometry plots of CD27 versus IgD expression on gated CD19^+^ B cells and CD27 versus IgM expression on gated CD19^+^CD27^+^ B cells are shown in S1A and S1B Fig, respectively.

Sorted B-cell populations were pelleted at 300g at 4°C, and finally flash frozen in liquid nitrogen before being stored at -80°C. Genomic DNA was purified from sorted B cell populations using the QIAmp DNA Blood Mini Kit (Qiagen). Genomic DNA was normalized and the equivalent of 50,000 cells was dispensed each of 188 wells of 96-well plates.

### PCR amplification and reduction of PCR bias

To amplify the CDR3 region of IgH, we used a 2-PCR reaction approach as previously described[23]. Briefly, the first step consists of a multiplex PCR that uses gene specific V-forward and J-reverse primers that bind to 47 V and 6 J functional genes as well as many of the pseudogenes for both V and J. The primers are designed for perfect complementarity to the germline V and J gene targets. In addition, the final five nucleotides of each primer were selected so as to bind to sequences that are much less likely to be affected by SHM[41]. The second PCR adds Illumina adaptor sequences and well-specific barcodes, for a total of 31 cycles of amplification.

Despite efforts to achieve consistent melting temperatures (T_m_) between all the V and all the J primers, there is a wide variation in amplification efficiency. To remove this bias, we created a synthetic set of IgH receptors with universal flanking sequences that allow for direct sequencing on the Illumina platform[40]. The synthetic genes include all V-J combinations labeled with barcodes that allow for the ready identification of each template. This synthetic immune system is sequenced directly to precisely determine the abundance of each template. Then, multiplex PCR amplification with the V and J gene primers is performed on the synthetic pool and the resulting DNA is also sequenced. Comparing the known starting abundances with the resulting amplified sequences, we are able to assess the relative amplification efficiency of each V and J primer. We then modify the concentration of the primers that over and under amplify. The process is iterated several times until the majority of the bias is removed. We have shown that the results of this process are robust to variations in the length, GC-content, and overall abundance of the template.

### Resolution of nucleotide sequences

To measure the amount of nucleotide assignment error in our analysis, we randomly selected molecules from the PCR amplified library of IgH receptor sequences, and sequenced them at a depth of at least 10 times the starting template quantity. In other words, since each well contained approximately 50,000 B cells, we aimed to sequence at least 500,000 molecules from each PCR library. This ensured that, even with some amplification variation and random sampling error, multiple copies of each template would be sequenced. Due to the very low error rate in Illumina sequencing (~.1%), the number of errors in a 130-basepair sequence is roughly distributed as *k*_*error*_ ∼ *Bin*(*n* = 130, *p* = .001), from which we compute Pr(*k*_*error*_ = 0) ≅ .88, and Pr(*k*_*error*_ = 1 | *k*_*error*_ > 0) ≅ .94. Thus, ~90% of all our templates result in no PCR or sequencing errors. Of the remaining ~10%, the large majority contain a single error. Given that these errors are not systematic, any particular error is almost always unique. Thus, we are able to readily correct these errors by identifying reads present once in the data set that differ by a single nucleotide from a sequence present multiple times, and collapsing them into the predominant clone. Additionally, since memory samples were found to have many more clones present in multiple wells, error correction was performed on data aggregated from all wells of a given sample. This ensures consistent consensus sequence assignment across wells. In terms of the diversity inference described below, this method of collapsing errors across wells is intrinsically conservative.

### Germline annotation of nucleotide sequences and SHM detection

The CDR3 region was identified according to the standard previously determined by the IMGT collaboration[9]. Identification of the V, D, and J gene segments was performed using a scored alignment across a definition list of all known V, D, and J gene and allele members from IMGT. The most likely assignments (allowing for ties for similar gene sequences) for each gene segment were then added to the sequence reads as their germline annotation. Somatic hypermutation was calculated over just the V gene segment, based on sequence variations from the assigned germline gene/allele match.

### Estimation of repertoire diversity from replicate occupancy data

To estimate clonal diversity, we derived an extension of an established sampling model in ecology and corpus linguistics: the Poisson abundance model[42–44]. This allows the construction of a likelihood function for replicate occupancy data parameterized by the richness and abundance distribution of the repertoire. Briefly, we synthesized the combinatorial probability of the replicate occupancy of a clone conditioned on sample abundance, with the Poisson abundance model of sample abundance conditioned on repertoire parameters. Analytically marginalizing over sample abundance as a latent variable, we formed the desired likelihood function and deployed tandem numerical and analytical optimizations facilitated by an asymptotic approximation for large richness. The full mathematical derivation and computational validation of this model can be found in the Supporting Information (S1 Method).

## Results and Discussion

### Immunosequencing of naive and memory B cells

In healthy adults, CD19^+^ B cells comprise 7–11% of lymphocytes circulating in peripheral blood[45]. This population is dominated by naive B cells, which correspond roughly to 65% of all peripheral B cells, while memory B cells account for about 30% of all circulating B cells[45]. To faithfully capture the breadth of the B-cell repertoire, we isolated naive (N, CD19^+^ CD27^-^ IgD^+^ IgM^+^) and memory (M, CD19^+^ CD27^+^) B cells from 400 mL of peripheral blood obtained from each of 3 healthy adult donors (D1, D2 and D3)[46]. Additionally, in order to estimate the reproducibility of the approach, we included two biological replicates of the naive B-cell sample from Donor 1 (i.e. D1-Na and D1-Nb).

These samples yielded 2–4 x 10^7^ naive B cells and 1.5–2 x 10^7^ memory B cells at greater than 97% purity from each donor. Considering that the approximately 5 L of peripheral blood of healthy adults is estimated to contain on average 6.5 x 10^8^ naive B cells and 3.0 x 10^8^ memory B cells[45], we calculate that by using a 400 mL sample, we captured 3.1–6.1% of the naive and 5–6.7% of the memory B cells circulating in peripheral blood, respectively.

Next, we sequenced a segment of the immunoglobulin heavy chain (IgH) gene from the naive and memory B cell populations purified from each donor that includes CDR3[18]. Since the CDR3 rearranges somatically during B cell development, the resulting sequences can be used to define unique B-cell clones, in the sense of descendants from a common naïve B cell; however, somatic hypermutation means that even among mature B cells that share a CDR3 by common descent, there can be additional sequence differences in e.g. the CDR1 and CDR2 regions.

**Fig 1:**
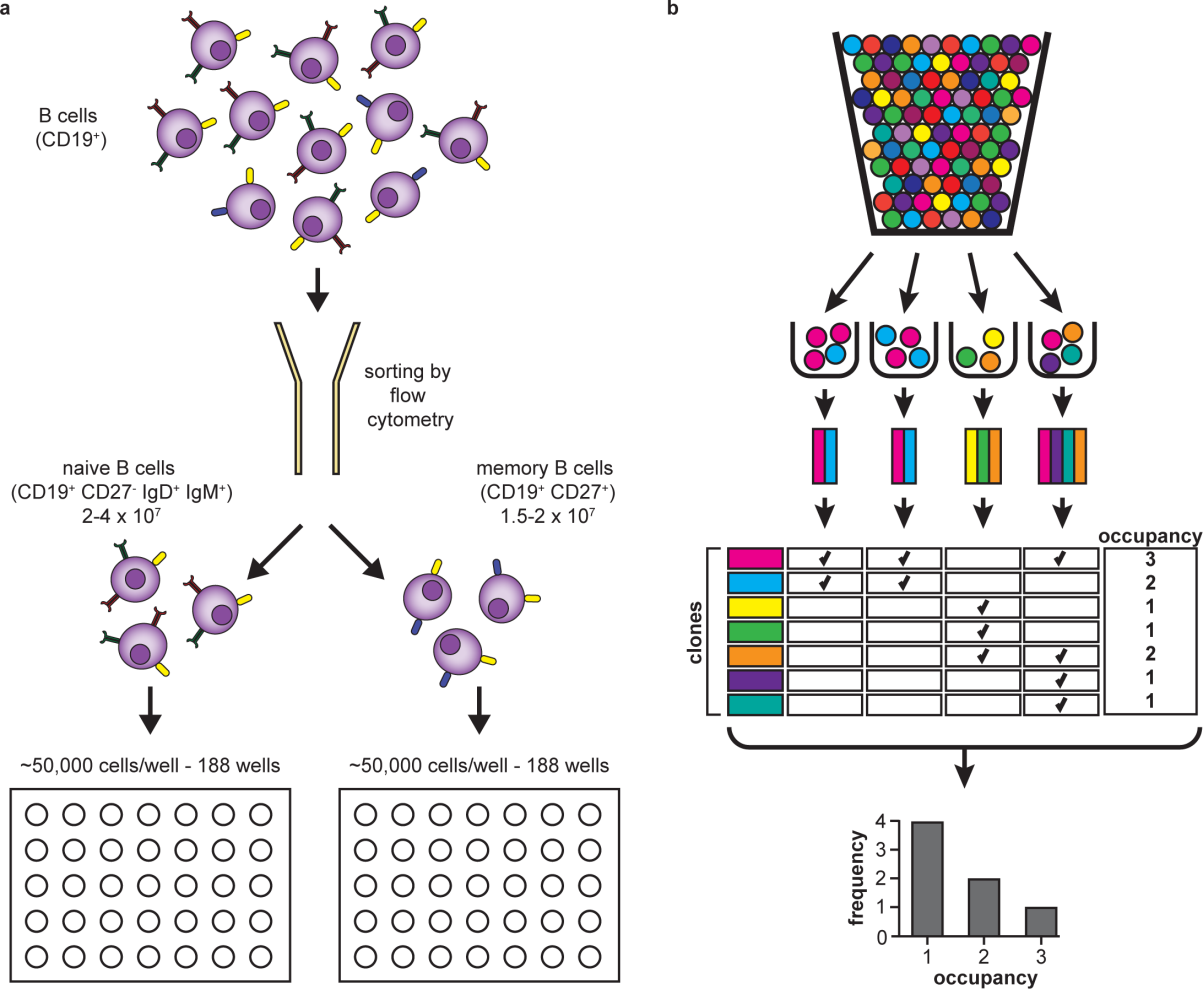
Experimental and informatic design. **(a)** Peripheral blood samples from three healthy donors were sorted using flow cytometry to isolate naive (CD19^+^ CD27^-^ IgD^+^ IgM^+^) and memory (CD19^+^ CD27^+^) B cells. For each sample, approximately 10^7^ cells were distributed into two 96-well plates (i.e., into 188 wells, resulting in ~50,000 cells per well), and processed by immunosequencing. **(b)** Schematic of the ‘urn sampling’ quantitation method. Cells are represented by colored balls, with each color indicating a different clone identity. Each ball (cell) is randomly allocated to a sample bin (well). Occupancy is calculated after censoring count information, and thus is expressed as presence or absence. The majority of clones are present in just one out of 188 wells, indicating that they were almost certainly represented by a single cell in the original sample.

In brief, for each of the samples, we extracted genomic DNA and we dispensed an amount corresponding to ~10^7^ naive or memory B cells into 188 wells of two 96-well plates (the remaining wells were used for controls). This resulted in the allocation of the equivalent of approximately 50,000 cells per well (Fig 1A). We then performed a two-step PCR, including a multiplex step that uses V and J-specific primers to amplify a region of the IgH gene, followed by a second amplification that adds unique well-specific barcodes and Illumina adaptors. Next, we used a HiSeq instrument to sequence a 130 nt-long segment of the IgH gene that includes the CDR3[18]. This approach enabled us to sample the naive and memory repertoires of B cells of three healthy individuals to a depth much greater than other studies.

The value of the resulting dataset depends both on the accuracy of the IgH nucleotide sequences and the quantitation of the abundance of each B cell clone. Importantly, there are two major obstacles that hinder the quantitative immunosequencing of IgH genes. The first challenge, which is shared by other immune genes such as those encoding for T-cell receptors, arises from the process of gene rearrangement and the resulting intrinsic diversity of both types of immune receptors. The second challenge, unique to B cells, results from the additional level of divergence from the genomic sequence generated by SHM in antigen-experienced cells. Our approach to address these challenges is described in the Material and Methods section, and our analytical approach is described in detail in the S1 Method included in the Supporting Information section.

In brief, we used a digital counting method that yields counts of clones based on their presence or absence in each of the 188 wells, as diagrammed in Fig 1B. Quantitative accuracy is achieved by inclusively sequencing the receptors in each uniquely-barcoded well. We aimed for a minimum of 10-fold coverage of each BCR molecule in each well, and achieved an effective coverage that ranged from 8 to 12 average reads per template in the different samples. We also analyzed the distribution of the number of unique productive BCRs over the 188 wells for each sample, as shown in S2 Fig. Most of the samples had an average of 40,000 unique productive rearrangements per well, with the exception of the naive sample from Subject 2, which had a lower number of unique productive rearrangements per well.

**Fig 2:**
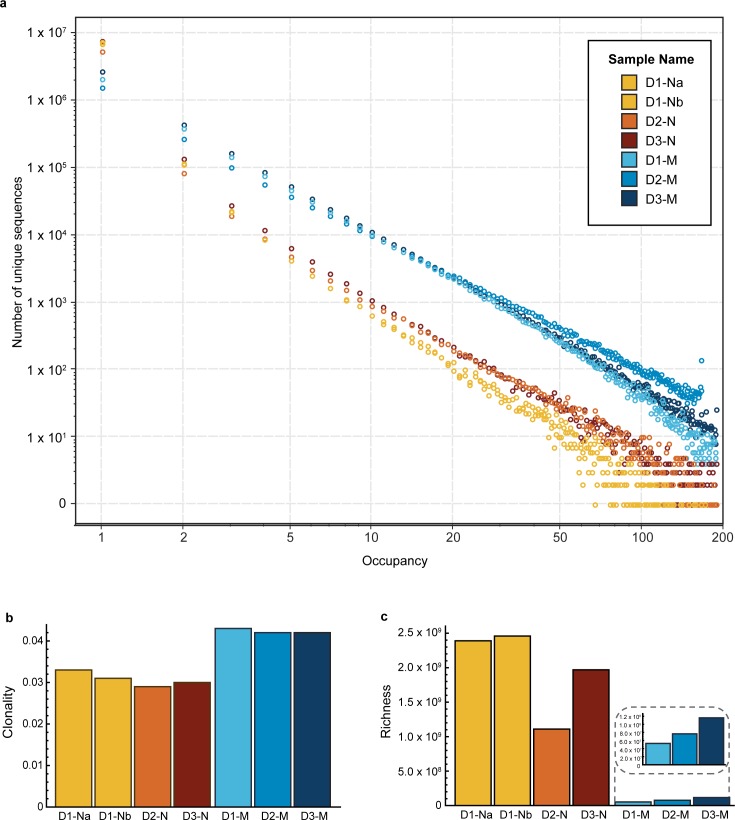
Inference of diversity in the naive and memory B-cell repertoires. **(a)** The graph shows the distribution of unique sequences, as the number of unique sequences (y-axis) versus their occupancy (x-axis) for the naive (orange) and memory (blue) samples for the three donors (D1, D2 and D3, including two technical replicates for the naive sample from Donor 1). The vast majority of the sequences have occupancy of 1. **(b)** Clonality index for all samples. **(c)** Richness index for all samples. While the clonality index is higher for memory samples, the richness index is higher for the naive samples.

Our method is binary, since we only consider presence or absence of each sequence in each well, and robust against a wide range of amplification efficiencies. The sequences in each well are identifiable by the presence of the unique barcode assigned to that well, and thus we report an “occupancy” value for each BCR sequence, which corresponds to the number of wells it was observed in. Clones with abundance in the repertoire of less than 1:1,000,000 B cells (i.e. the vast majority of all B-cell clones) will rarely be present more than once in any well. Therefore, for most B cells, their sample abundance will be equal to the number of wells they are observed in. We determined that the vast majority of clones have an occupancy value equal to 1 (Fig 2A). Since multiple cells of the same clone are unlikely to appear in any given well, this strongly implies that a single cell out of the initial 10^7^ expressed that particular BCR sequence. As occupancy increases, this metric becomes a decreasingly precise (and increasingly negatively biased) estimator for sample abundance, since the incidence of multiple occurrences of a given clone in a single well becomes more probable.

### Diversity of the naive and memory B cell receptor repertoires

We first compared the overlap between the naive and memory B-cell repertoires of the three donors studied (Table 1). For this analysis, we only considered exact sequence matches.

**Table 1:** Overlap among the naive and memory repertoires of the three donors.

		1			2		3	
		Na	Nb	M	N	M	N	M
1	Na		4.18E-03	7.87E-04	4.41E-04	8.34E-05	6.96E-04	1.07E-04
	Nb	4.55E-03		8.21E-04	4.44E-04	8.42E-05	6.92E-04	1.08E-04
	M	1.75E-03	1.68E-03		5.42E-05	9.48E-06	8.90E-05	2.03E-05
2	N	6.55E-04	6.06E-04	3.61E-05		2.38E-03	7.06E-04	1.15E-04
	M	2.42E-04	2.25E-04	1.24E-05	4.67E-03		2.65E-04	5.43E-05
3	N	6.66E-04	6.09E-04	3.83E-05	4.56E-04	8.74E-05		4.07E-03
	M	1.94E-04	1.80E-04	1.65E-05	1.40E-04	3.38E-05	7.70E-03	

For each sample obtained from each of the donors (D1-Na, D1-Nb and D1-M; D2-N and D2-M; and D3-N and D3-M), the table indicates the pairwise overlap between repertoires, computed as the fraction of the unique sequences for each sample in the rows labeled to the left that are also found (with no mismatches allowed) in the each of the samples listed in the columns. The color gradient of the cells indicates the degree of overlap, with higher overlaps indicating a darker shade of red.

Due to the intrinsically large size and diversity of the B-cell repertoire, the overall overlap between samples is small. However, as expected, it is higher between the two independent replicates of the naive repertoire of Donor 1 than between those of different donors. Also, the naive and memory B-cell populations of each donor are more similar to each other than to those of different donors.

Next, for each sequence present in the data we computed the maximum well occupancy among all samples (a measure of clonal abundance), and also the number of subjects the sequence was observed in. S3 Fig shows the distribution of maximum occupancy among sequences found in only 1 subject, in any two subjects, and in all three subjects. We observe that shared sequences (those present in two or three subjects) tend to have higher maximum occupancy. This could be the result of shared memory cells resulting from common pathogen exposures among subjects, or alternatively, the consequence of recurrent generation of high-probability V(D)J recombinations that are identical by state but not by descent in different individuals.

We also estimated the clonal diversity of the repertoires–i.e. the number of distinct somatically rearranged receptors present in each repertoire and their relative abundances–which defines the search space available for immune recognition and is therefore essential for the quantitative characterization of the BCR repertoire. For each sample, we inferred two diversity indices: *richness*, defined as the number of distinct clones, and *clonality*, a measure of abundance uniformity that ranges from 0 (maximally uniform) to 1 (most disparate, or clonally dominated; see the Materials and Methods and S1 Method for a detailed description of these indices). Fig 2B shows the maximum likelihood estimates of clonal diversity. Using either diversity metric, the samples cluster distinctly by cell type, and these results were consistent across individuals. As expected, our results indicate that memory clones have more disparate repertoire abundances (higher clonality) than naive clones, and that naive clones are extremely diverse.

Our replicate PCR well methodology accurately assesses the abundance of nearly all B-cell clones in each sample. A small number of memory clones are present at high frequency, and thus are found in all or nearly all of the replicate PCR wells. This is expected to cause negative bias in the clonality inferences for the memory populations. Despite this conservative bias, the memory and naive populations cluster distinctly.

The inferred richness of the naive B-cell repertoire is of a similar magnitude to the expected abundance of naive B cells in the peripheral blood (~1x10^9^)[45], suggesting that the typical naive clone does not undergo proliferation prior to antigen encounter. In contrast, the richness of the memory B-cell population is consistent with each clone undergoing several divisions on average. The relatively higher clonality observed for memory cells as compared to naive cells indicates that a small percentage of these clones experience significant proliferation. Our conclusion that the typical naive B-cell clone undergoes no proliferation prior to antigen encounter raises questions regarding previous calculations that suggested that naive B cells in the peripheral blood of adults undergo approximately 1.9 cycles of homeostatic proliferation on average[47]. However, it is important to point out that the study by Van Zelm *et al*. uses an indirect method of estimating the replication history based on deletion circles, and that, unlike our approach, it does not have the ability to resolve distinct clones. On the other hand, we do not measure replication history and instead calculate it from the diversity metric and estimates of the number of B cells in the periphery reported in the literature. Thus, both sets of results are not directly comparable and do not necessarily contradict each other.

In summary, our data confirm that the naive repertoire of a healthy adult is extremely rich, and thus suggests that the typical naive B-cell clone undergoes no proliferation prior to antigen encounter, while we observe that memory B-cell clones undergo several cycles of division on average. Future studies will focus on mining this extremely deeply-sequenced data to further understand ongoing maturation of clones within the memory compartment at steady state. The assay also has the potential to determine whether the different subsets of cells contained in the memory compartment (i.e. switched memory cells, unswitched memory cells, as well as any plasmablasts or plasma cells present due to ongoing immune responses) possess different distributions of mutation rates.

### Examples of possible explorations of this dataset

To demonstrate that our data are accurate and of high quality, we made use of these tools to answer several fundamental questions about the B-cell repertoire in healthy individuals. In addition to the clonal diversity inferences described above, we provide a set of four examples that illustrate the utility of the data set and the related analysis tools. For each of these examples, we created a dashboard in the immunoSEQ Analyzer workspace (http://adaptivebiotech.com/link/publicBCellResource) so that the analysis of each example and the accompanying visualizations that follow can be reproduced by the user.

#### Example 1: Characterization of IGHV family and gene usage

The IGH V locus contains over 50 functional genes (depending on the individual’s haplotype) that are classified into 7 families based on nucleotide sequence homology[48]. Each gene segment has a certain likelihood of undergoing rearrangement and being incorporated into a mature immunoglobulin molecule, and in addition the process of negative selection of immature B cells further restricts V gene segment use, resulting in an unequal representation of V gene families in the naive B-cell repertoire. Similarly, the positive selection of naive B cells to populate the memory compartment results in variations in V gene segment representation[13, 49].

**Fig 3:**
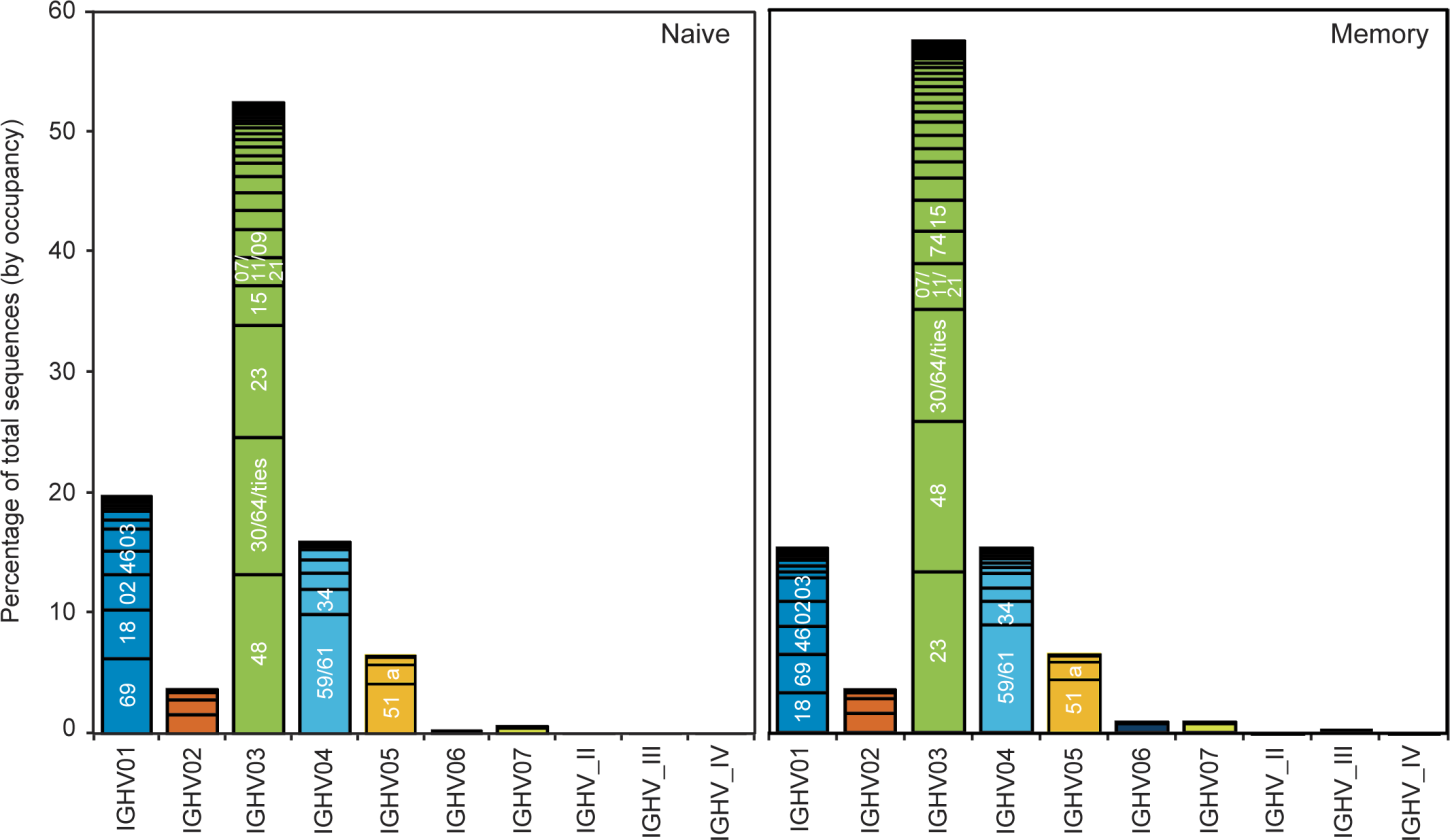
V family and V gene usage patterns. The histograms show the relative percent of total sequences (by occupancy) for each of the IGHV families (as shown under the graphs), for the naive (left panel) and memory (right panel) samples, aggregated for the three donors. Within each family, discrete bands represent each of the individual genes. The most abundant genes within each family are indicated (e.g., 69 in IGHV01 refers to the gene IGHV01-69). Overall, memory samples contain fewer IGHV01 and more IGHV03 family sequences than naive samples, with some gene-level differences evident as well.

Traditionally, standard measurements of TCR usage in T cells have utilized PCR-based V beta spectratyping (reviewed in reference [7]), but no equivalent approach exists for the analysis of V gene usage in B cells. However, recent immunosequencing approaches have begun to shed light on B-cell gene usage[18, 19]. To assess the broad similarities and differences in gene usage between the naive and memory B-cell repertoires, we compared the IGHV family and gene usage in naive and memory B cells in three healthy donors (Fig 3). In agreement with previous reports, we found that the IGHV3 gene family is utilized most commonly in both repertoires[49, 50]. Moreover, we observed that, in these subjects, IGHV3-48 is the most commonly used V gene in the naive repertoire followed by IGHV3-30 or IGHV3-64, two genes that are indistinguishable over the region covered by the sequence reads. In the memory repertoire, IGHV3-23 is used most commonly, followed by IGHV3-48. We found that the second most commonly expressed gene family in the naive repertoire of these subjects corresponds to IGHV1, followed by IGHV4. In contrast, the memory repertoire has equivalent representation of the IGHV1 and IGHV4 gene families. At the gene specific level, we observed a decrease in the relative frequency of IGHV1-69 and IGHV1-18 within the IGHV1 family in memory compared to naive B cells, consistent with previous studies[13]. Taken together, these data, which were obtained from a single experiment, reproduce observations from several previously published studies [13, 18, 49–51], validating the utility of this dataset.

#### Example 2. Measurement of CDR3 length distribution

**Fig 4:**
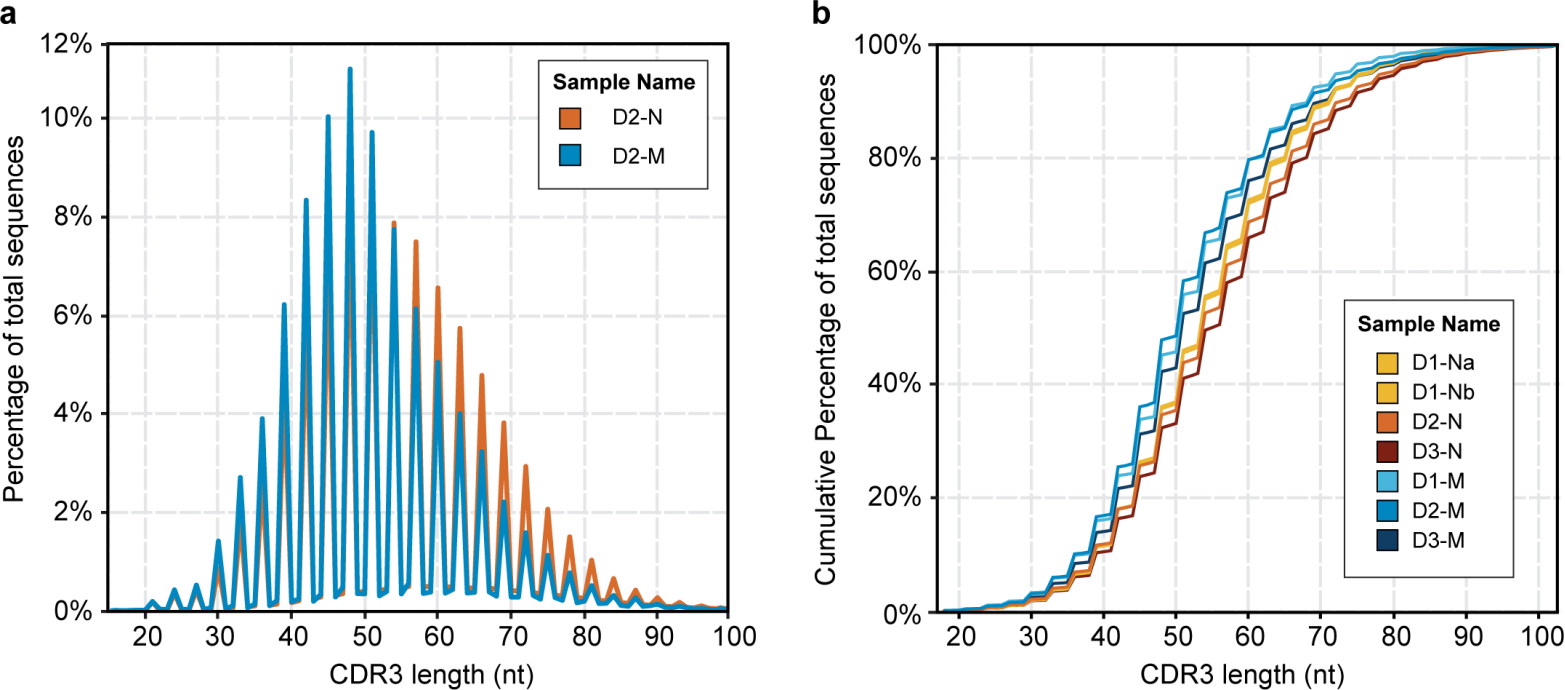
Comparison of CDR3 lengths in naive versus memory B-cell samples. (**a**) The graph shows the normalized percentage of total sequences for the naive (orange) and memory B cells (blue) from donor D2. (**b**) The graph shows the cumulative percentage of total sequences at a given CDR3 length for all naive and memory samples, as indicated in the inset. The technical replicates for donor D1 overlap closely and are not distinguishable in this figure. The memory repertoire is consistently 3 nucleotides (or 1 amino acid) shorter than the naive repertoire at the same cumulative frequency.

The immunoglobulin CDR3 is the most important determinant of antibody-antigen recognition[52, 53]. Its length varies mostly due to recombination, and can also change slightly from SHM. Therefore, we compared the CDR3 length distribution of the naive and memory repertoires to understand both the limits and flexibility of the antigen-binding capacity of B cells. We found the average CDR3 length in the naive B-cell repertoire to be 48 nucleotides, while the memory B cells had, on average, a CDR3 length of 45 nucleotides (Fig 4). Unproductive CDR3 sequences have an even longer average size (~60 nt) than that seen for productive sequences in naive or memory cells. These two facts suggest that, while the B-cell recombination process generates long and highly diverse CDR3 regions, functional clones that become part of the memory repertoire are biased towards shorter CDR3 sequences. In addition, we observe that there is a greater variability in CDR3 length in naive cells compared to memory cells, suggesting that the naive repertoire has the potential to bind a wider range of antigens than are actually encountered by the donors in this study. These data agree with previous findings [13, 18, 54], further confirming the validity of our dataset.

#### Example 3: Assessment of purity of flow-cytometry sorted cell populations

**Fig 5:**
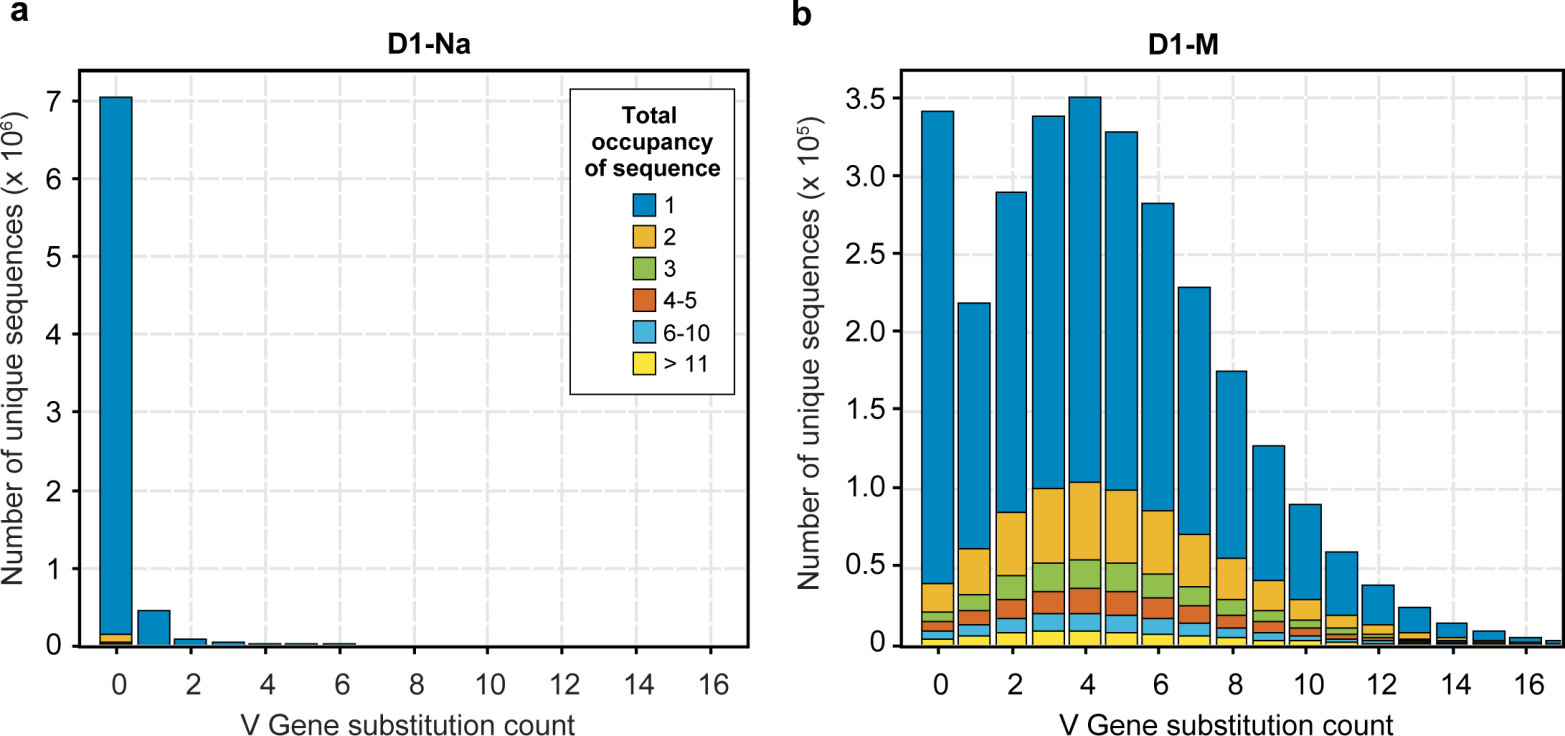
Comparison of Somatic Hyper Mutation in paired naive and memory B-cell samples from the same donor. The figure shows data for the naive (**a**) and memory sample (**b**) from Donor 1, which is representative of all three donors. The x-axis corresponds to the number of substitutions differing from the germline V gene sequence, and the y-axis indicates the number of unique sequences that display that number of substitutions. The colors indicate different total well occupancies, with blue indicating singletons present in just one well, and the other colors showing progressively higher well occupancy, as indicated in the figure. The majority of the sequences in the naive B-cell sample have 0 substitutions and correspond to low abundance clones observed in a single well (blue). In contrast, the memory B cell sample from the same individual shows a much broader distribution of substitutions, as well as many more sequences with occupancy greater than 1.

Since SHM occurs during antigen-induced maturation, a naive B cell is characterized by the absence of substitutions in its germline V gene[5]. Thus, to examine the purity of our sorted B-cell populations, we determined the rate of substitutions in the V genes of the naive and memory B-cell repertoires (Fig 5). Approximately 95% of sorted naive B cells displayed no V gene substitutions, and had low clonal abundances, which are typical of naive cells. In contrast, memory B cells harbored an average of 3–4 substitutions per 100 nt in the V genes, and additionally displayed a much broader range of clonal abundances, as expected of antigen-experienced B cells. Taken together, these analyses suggest that our method accurately and faithfully captures the circulating B-cell populations.

#### Example 4: Analysis of somatic hypermutation in memory B cells

Affinity maturation, including somatic hypermutation and class switching, is critical to the production of functional antibodies[55–60]. We were able to easily define somatic hypermutation sites by identifying variations from germline sequences within the sequenced region of the V gene. While a certain number of single nucleotide variations in the V gene may result from inherited SNPs, a review of the V gene sequences observed in naive cells in the same individual makes it easy to exclude this possibility in most cases.

**Fig 6:**
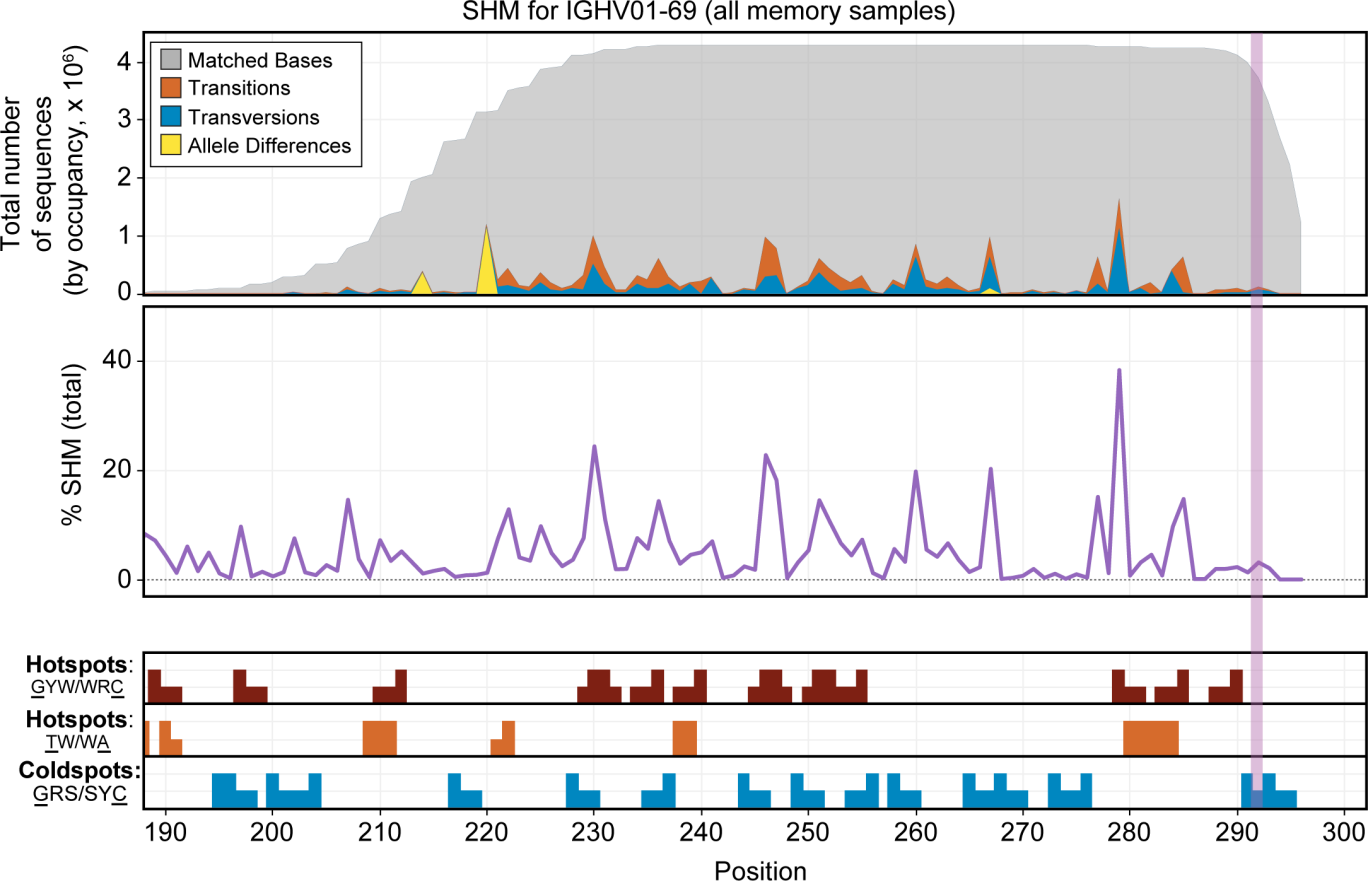
Somatic hypermutation pattern observed over the sequenced region of the IGHV01-69 gene. The figure includes combined data from the memory B-cell population for all 3 donors. The top panel shows the total distribution of sequenced bases by occupancy for the primary allele of IGHV01-69. Nucleotides that match the germline sequence are displayed in gray. Transitions are shown in orange and transversions in blue. Allelic differences, which are also seen in the naive samples, are indicated in yellow. The vertical dotted line marks the average start of the CDR3 region. The middle panel shows the normalized percentage SHM by base for this gene across the memory B cell samples for all three donors. The bottom panel shows suspected SHM hotspot (red and orange bars) and coldspot (blue bars) motifs present in the sequence of this gene over the region assayed. Positions with higher bars indicate bases targeted within the motif (underlined in the legend to the left). The GYW/WRC pattern (red) explains most of the significant sites of SHM for this gene, but some spots of high mutation are not captured by the displayed motifs. In the data viewer, this view can be generated for any V gene and for any combination of data sets.

After identifying likely somatically hypermutated residues in the V gene segments, we created a set of tools to view these data for all genes and samples over the sequenced V gene region. Fig 6 shows an example of the resulting data for gene IGHV1-69. Our analysis and visualization tools allow a clear visualization of SNPs and transition/transversion rates (top panel), as well as overall SHM rates by position (middle panel) gleaned from our very deep sampling of memory B-cell sequence data. In addition, several reported hotspot and coldspot AID targeting motifs[61] can be evaluated (bottom panel). The most frequently reported hotspot motif (most generally described as GYW/WRC on the two strands[6]) accounts for many of the observed positions with high SHM levels, while some nucleotides that display SHM, such as nucleotide 267 in several V genes including V01-69 and V03-23, are not part of a known hotspot motif. It is possible that mutations of this position, which flanks the CDR3, might have increased functional importance for improved antibody binding, despite the absence of known AID-targeting motifs.

## Conclusions

In this study, we provide the research community with an accurate and rich dataset of BCRs, as well as a set of straightforward tools to enable its in-depth study. By combining flow cytometry purification of peripheral B cells with high-throughput immunosequencing of 10 million naive and 10 million memory B cells from each of three healthy adult donors, we generated a BCR sequence library containing more than 37 million unique BCR sequences. Whereas some of the currently existing databases, such as IMGT[9], contain a large number of curated IgH sequences from many individuals, this method allowed us to probe the B-cell repertoire of a small number of individuals at an unprecedented depth. In parallel, we developed set of tools tailored to analyze and visualize the resulting data set, which can be accessed from http://adaptivebiotech.com/pub/robins-bcell-2016 (please follow the ‘Advanced Visualizations’ link).

As an example of the utility of our dataset, we assessed a fundamental property of the BCR repertoires, i.e. their clonal diversity. To do this, we approximated high throughput digital cell counting using a multi-replicate experimental design, and we inferred the clonal diversity of the memory and naive BCR repertoires of three healthy adults using a novel likelihood model.

To further illustrate the utility of these data and the associated tools, we present several other examples that assess general properties of B-cell repertoires that have been previously investigated at a smaller scale, including V gene family usage patterns; the length of CDR3 regions; the numbers of SHM substitutions, and the patterns and types of SHM in naive and memory B cells. Importantly, our observations match previous reports and thus confirm the robustness of our dataset.

Finally, the many-replicate experimental design employed in this study, in which each of the 188 PCR wells corresponds to a replicate sample, constitutes a sample abundance probe robust to the inherent stochasticity of PCR amplification. Moreover, this approach represents a crucial quantitative advance over previous sequencing studies of antigen receptor repertoire diversity, which have been limited by either poor quantitation or by the lower throughput of single-cell methods[27, 62, 63]. We expect that these data will be used by other experts in the field of immunology to address additional fundamental questions about BCR development and *in vivo* antigen binding in humans.

### Data Availability

Access to the data set resulting from the experiments described in this study (both at the well level and at the sample level), as well as a link to the tools we developed to enable the analyses presented herein, can be found at http://adaptivebiotech.com/pub/robins-bcell-2016. We have also assigned a unique identifier to this dataset: http://doi.org/10.21417/B71018. The immunoSEQ Analyzer interface includes several tools that can be used to perform further analyses of the data. The “Advanced Visualization” link found in the landing page for this dataset enables access to Fig 2 to Fig 6 in this study, and each of them is followed by a set of interactive dashboards that allow viewing different aspects the data, such as Occupancy (data underlying Fig 2), VDJ tools (data underlying Fig 3), CDR3 tools (data underlying Fig 4), Substitutions tools (data underlying Fig 5), and SHM tools (data underlying Fig 6). Most dashboards include a sample selection option: data are coded by sample type (naive vs. memory) and for each of the three donors studied (including the two repeats for the naive sample from donor 1). Several of the dashboards include filters that allow viewing subsets of the data (e.g. sequences for productive vs. non-productive rearrangements, out-of-frame sequences or sequences with STOP codons). The code for the tools developed for the analysis can be downloaded from the Public B cell dataset code link.

Finally, the full dataset can also be downloaded from the Public B cell dataset link, as well as from the Dryad Digital Repository at http://datadryad.org/resource/doi:10.5061/dryad.35ks2.

## Supporting Information

S1 Fig Representative contour plots of peripheral blood B-cell subsets. (PDF)

S2 Fig Distribution of the number of unique sequences across 188 wells for each sample used in this study. (PDF)

S3 Fig Distribution of maximum occupancy among sequences found in only 1 subject, in any two subjects, and in all three subjects. (PDF)

S1 Method Replicate immunosequencing as a robust probe of antigen receptor repertoire diversity. (PDF)
