# Supplementary material for: A Public Database of Memory and Naive B-Cell Receptor Sequences
Source: PLoS One. 2016 Aug 11;11(8):e0160853. doi: 10.1371/journal.pone.0160853 (PMC4981401; doi:10.1371/journal.pone.0160853)
Supplement: S1 Fig — (PDF) [file pone.0160853.s001.pdf]

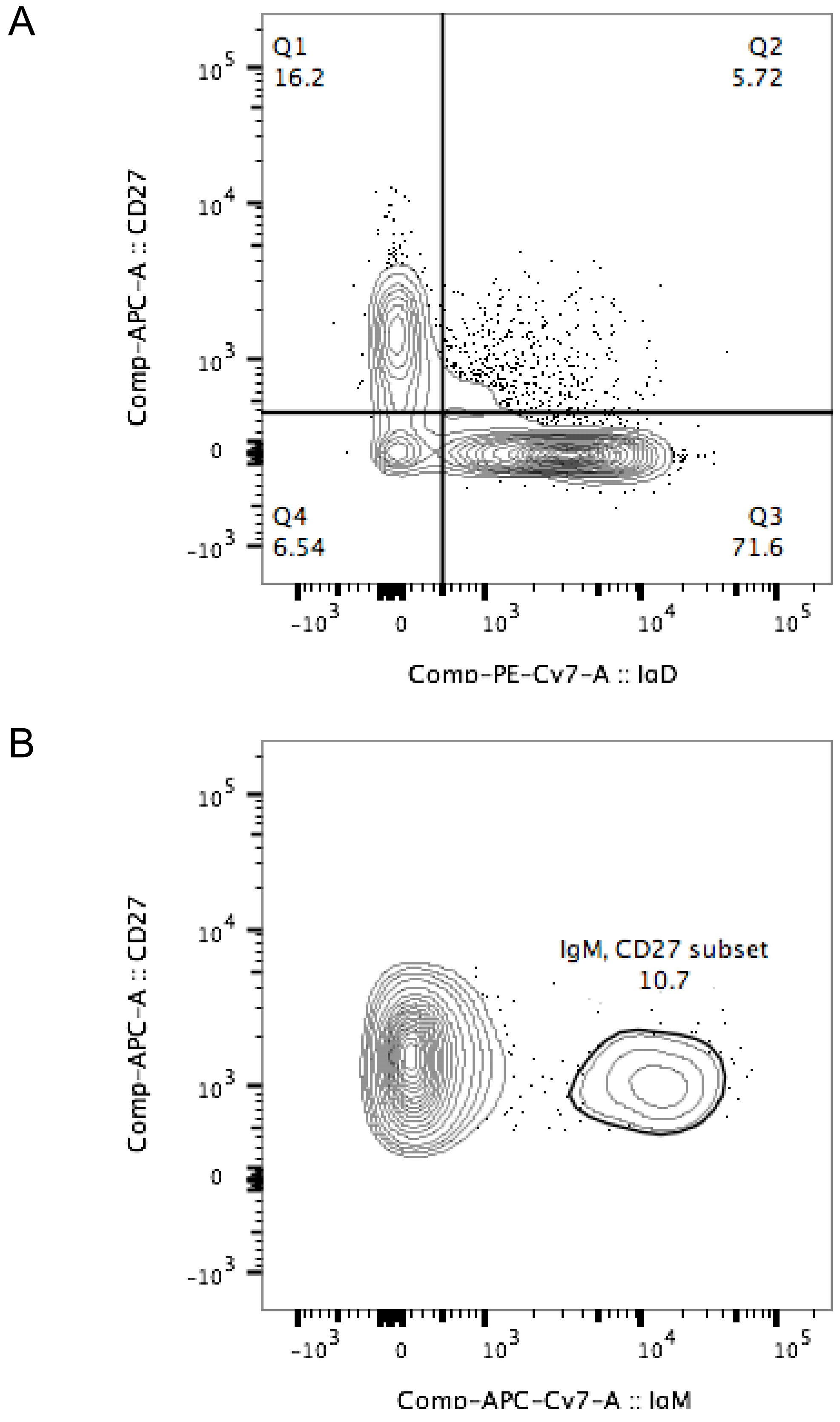

S1 Fig: **Representative contour plots of peripheral blood B-cell subsets.** (**a**) CD27 (y-axis) and IgD (x-axis) expression on gated $CD19^{+}$ B cells. (**b**) CD27 (y-axis) and IgM (x-axis) expression on gated $CD19^{+}CD27^{+}$ B cells.
