# Supplementary material for: A Public Database of Memory and Naive B-Cell Receptor Sequences
Source: PLoS One. 2016 Aug 11;11(8):e0160853. doi: 10.1371/journal.pone.0160853 (PMC4981401; doi:10.1371/journal.pone.0160853)
Supplement: S2 Fig — (PDF) [file pone.0160853.s002.pdf]

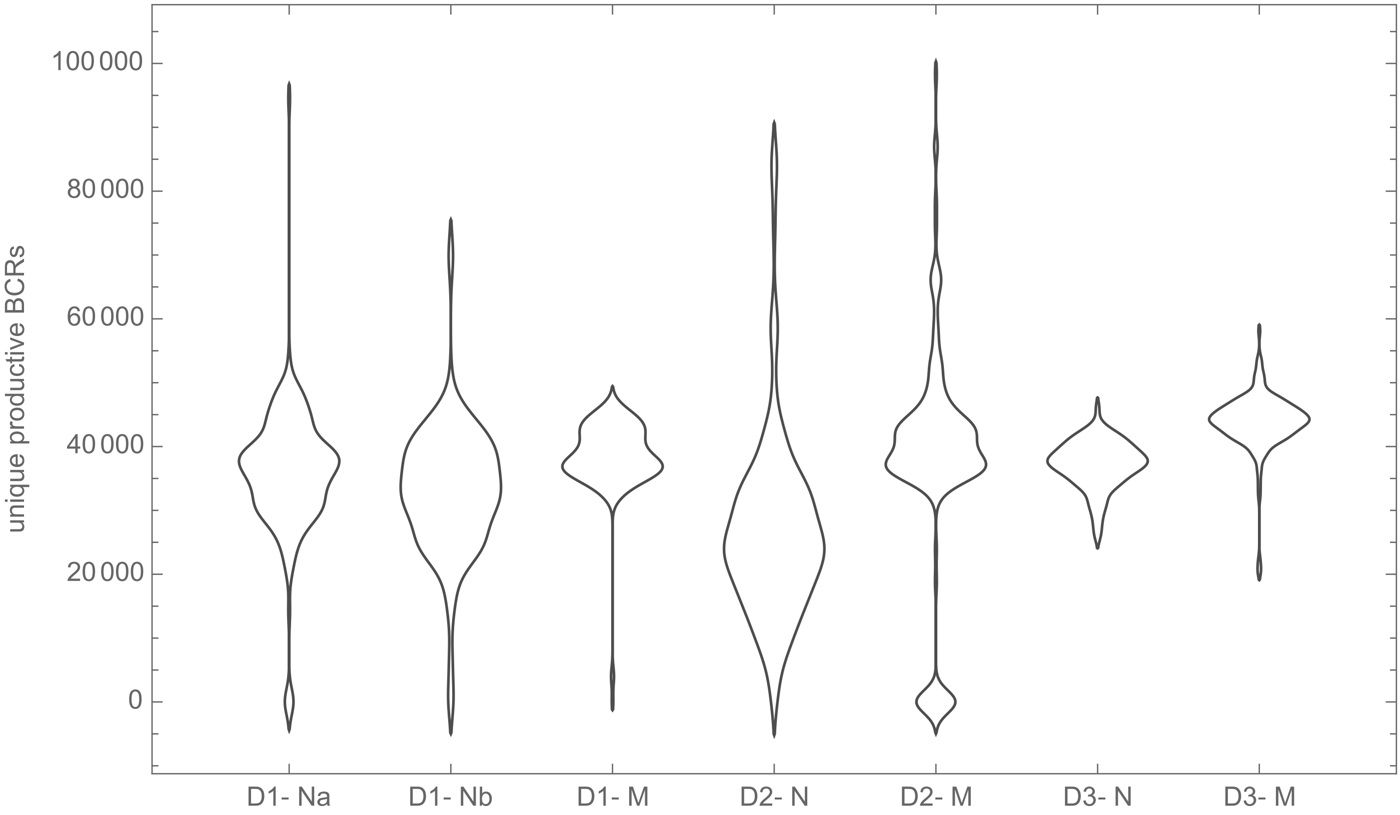

S2 Fig: **Distribution of the number of unique sequences across 188 wells for each sample used in this study.**
