# Supplementary material for: A Public Database of Memory and Naive B-Cell Receptor Sequences
Source: PLoS One. 2016 Aug 11;11(8):e0160853. doi: 10.1371/journal.pone.0160853 (PMC4981401; doi:10.1371/journal.pone.0160853)
Supplement: S3 Fig — (PDF) [file pone.0160853.s003.pdf]

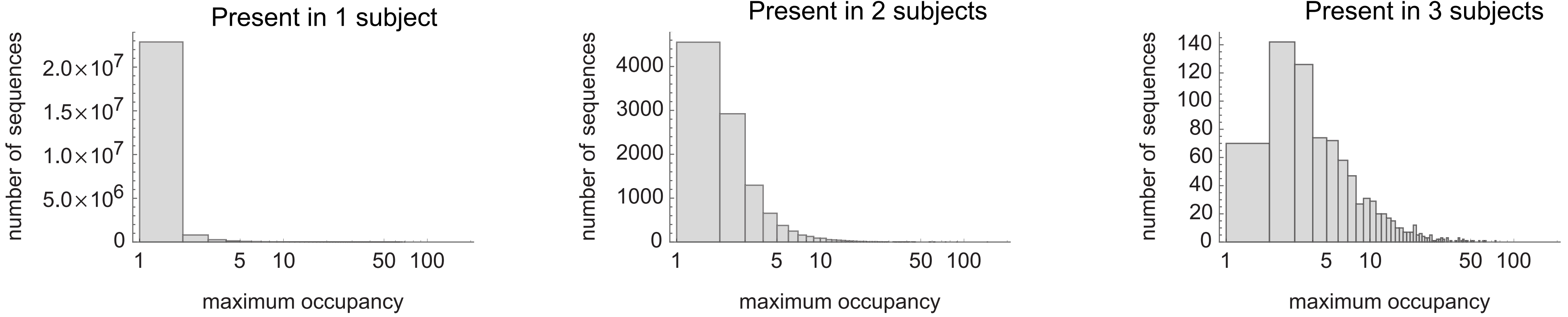

S3 Fig: **Distribution of maximum occupancy among sequences found in only one subject, in any two subjects, and in all three subjects.**
