# Supplementary material for: A Public Database of Memory and Naive B-Cell Receptor Sequences
Source: PLoS One. 2016 Aug 11;11(8):e0160853. doi: 10.1371/journal.pone.0160853 (PMC4981401; doi:10.1371/journal.pone.0160853)
Supplement: S1 Method — (PDF) [file pone.0160853.s004.pdf]

Supplementary Method:
Replicate immunosequencing as a robust probe of antigen receptor repertoire diversity

I. INTRODUCTION

Previous approaches to antigen receptor repertoire diversity estimation redeploy methods developed in the ecology and corpus linguistics literature to estimate species diversity and vocabulary size (see review [1]), respectively. Specifically, Poisson abundance models, with both parametric and nonparametric estimators, are used. Although conceptually erroneous, mark-recapture formulae have also been applied [2]. Antigen receptor repertoires more closely achieve the idealizations of these models than the their original applications; populations are very large and well-mixed, and detection probabilities are homogeneous. However, studies suffer from limitations in sequencing data that blunt sophisticated computational approaches.

Robins et al. [3] assessed T cell receptor (TCR) richness from high-throughput immunosequencing data using a nonparametric empirical Bayes method requiring divergent series regularization [4, 5] (a substantially improved regularization technique, applied to estimating the molecular complexity of PCR libraries, is advanced in [6]). However, the sequencing read count assigned to each unique TCR (after error correction) was associated with its clonal abundance in the sample. This introduces noise and bias, since each single template is stochastically amplified by PCR. Although this high-throughput study captured the diversity of a realistic biological sample, inference of repertoire richness was problematic due to limited quantitation of sample abundance for each clone.

Rempala et al. [7, 8] employed a likelihood model and posterior inference for mouse TCR richness using single-cell sequencing to quantitate sample abundance of T cell clones. Although this approach allows for precise quantitation of sample abundance, it is so low throughput (one cell per well on a 96-well plate) that diversity estimation was only possible for transgenic mice engineered to have dramatically limited TCR diversity. Although quantitatively principled, severe experimental limitations restricted the study to less biologically relevant repertoires.

II. EXPERIMENTAL DESIGN

In the present study a high-throughput and quantitatively robust (albeit indirect) probe of B cell clone sample abundance was devised. B cells from three adults were sorted into memory and naive populations (with two naive replicates for subject 1), each with $\sim 10^7$ cells (Fig. 1a, main text). Extracted DNA from each sample was evenly partitioned into 188 PCR replicates for amplification and uniquely barcoded for immunosequencing of the rearranged IgH locus [9], identifying clones by unique CDR3 sequence in their B cell receptor. Instead of relying on sequencing read counts to estimate a clone's sample abundance, we use its occupancy - the number of replicates it is observed in. In the regime of small occupancies, this approximates digital cell counting - a clone observed in only one replicate almost surely has a sample abundance of one cell. For larger sample abundances, co-occupancies become more probable, so occupancy increasingly underestimates abundance. Clones with sample abundance much larger than the number of replicates will saturate, appearing in all replicates.

To address possible template quantity variation across replicates and non-detection effects, we selected the subset of 150 replicates for each sample having minimum variance in the number of unique clones. Removing replicates with outlying allocations of cells or underperforming amplification is necessary to avoid breaking exchange symmetries invoked in our model.

III. MODEL

We advance a combinatorial extension of a well-studied model of sample abundance, enabling application to occupancy data. After introducing a parameterization of this extended model, a maximum likelihood diversity estimation is introduced, validated with simulations, and applied to BCR repertoire occupancy data to infer both richness (the number of clonal species) and an index of relative diversity (evenness of clone abundances).

A. Poisson abundance model of replicate occupancy

As is canon in the ecology and corpus linguistics literature, we begin by modeling sampling from a diverse population as a superposition of homogeneous Poisson processes. A mixing measure, $\mu(\lambda)$, characterizes the distribution of Poisson rates over all categories (B cell clones, as identified by productively rearranged IgH CDR3 segment, in our case). Since a clone's Poisson rate, λ, is given by its repertoire fraction times the number of cells sampled, $\mu(\lambda)$ is tantamount to the repertoire clonal abundance distribution. Homogeneity entails the approximation that the repertoire is effectively an infinite reservoir (or is being sampled with replacement), such that the data is not sensitive to depletion of the population fractions of the sampled clones. An equivalent urn model samples with replacement from a finite urn with an unknown number of ball colors, or without replacement with an urn with an infinite number of balls and

specified fractional abundances for each color. The total number of balls (cells) sampled from the urn (repertoire) is taken to be a Poisson sample from a multinomial population. The marginal distributions of sample cellular abundance, j, of each clone are then independently and identically distributed as

$$p\left(j|\mu(\lambda)\right) = \int_0^\infty d\mu(\lambda)\frac{\lambda^j e^{-\lambda}}{j!}$$

To model replicate occupancy, we assume that each sampled cell is randomly assigned to one of L possible replicates with equal probability (Fig. 1b, main text). Because the sample material was partitioned equally among the L replicates, it is not strictly correct to assume that each cell is assigned to a replicate independently. However, for large samples this approximation is very accurate. If the sample contains N cells, then under this model the number of cells in each replicate is binomially distributed with N trials and success probability $1/L$. The coefficient of variation is $\sqrt{(L-1)/N}$. The number of replicates used in this study was 150, and about 10 million cells were sequenced for all samples, leading to a coefficient of variation of about 0.004.

The distribution of a clone's replicate occupancy, i, conditioned on sample abundance, j, is then determined combinatorially as

$$q\left(i|j\right) = \frac{\binom{L}{i} i! \left\{ {j \atop i} \right\}}{L^j}.$$

This is simply the ratio of the number of ways to partition j cells into i out of L replicates, divided by the total number of ways to allocate j cells among L replicates. $\left\{ {j \atop i} \right\}$ denote Stirling numbers of the second kind, which count the number of ways to partition j distinguishable objects into i indistinguishable nonempty subsets.

Marginalizing over the hidden sample abundance gives the distribution of each clone's occupancy as

$$\begin{aligned} r\left(i|\mu(\lambda)\right) &= \sum_{j=0}^{\infty} q\left(i|j\right) p\left(j|\mu(\lambda)\right) \qquad (1) \\ &= \sum_{j=0}^{\infty} \frac{\binom{L}{i} i! \left\{ {j \atop i} \right\}}{L^j} \int_0^\infty d\mu(\lambda)\lambda^j e^{-\lambda}/j! \\ &= \binom{L}{i} i! \int_0^\infty d\mu(\lambda) e^{-\lambda} \sum_{j=0}^{\infty} \frac{\left\{ {j \atop i} \right\}}{j!}\left(\frac{\lambda}{L}\right)^j \\ &= \binom{L}{i} \int_0^\infty d\mu(\lambda) e^{-\lambda}\left(e^{\frac{\lambda}{L}}-1\right)^i, \end{aligned}$$

where we have exchanged the order of integration and summation, and identified the sum as a well-studied exponential generating function for the Stirling numbers ([10], p.83). In formal power series notation, $\left\{ {j \atop i} \right\} = j! \left[z^j\right]\left((e^z-1)^i/i!\right)$. We consider a finite-dimensional subspace of measures, $\mu_\theta(\lambda)$, parameterized by the vector θ, and thus write (1) as

$$r_\theta(i) = \binom{L}{i} \int_0^\infty d\mu_\theta(\lambda) e^{-\lambda}\left(e^{\frac{\lambda}{L}}-1\right)^i. \qquad (2)$$

For a repertoire with clonal diversity S, the sample occupancy of each clone is drawn from distribution (2). Let $l_1, l_2, \ldots, l_S$ denote the replicate occupancies of S labelled clones. For a very diverse repertoire and a limited sample, many clones will not be sampled, and thus have occupancy zero (the *missing species*). Due to exchangeability of the clone labels, it is sufficient to consider the frequencies of nonzero occupancies, defined by the vector indicator random variable $o = (o_1, o_2, \ldots, o_L)$, with $o_i = |\{c \in \{1, 2, \ldots, S\} : l_c = i\}|$ (the number of clones occupying exactly i replicates).

We may write a multinomial likelihood function as

$$\mathcal{L}\left(\theta, S|o\right) = \frac{S!}{(S-s)!} r_\theta(0)^{S-s} \prod_{i=1}^{L} \frac{r_\theta(i)^{o_i}}{o_i!} \qquad (3)$$

where $s = \sum_{i=1}^{L} o_i$ is the sample diversity. There are $S - s$ missing species.

B. Parameterization

Antigen receptor repertoires have been observed to follow Zipf's law [11]: the logarithms of the frequencies of clones are inversely proportional to the logarithms of their ranks by frequency. As a continuous analog of this discrete power law behavior, we make the parametric ansatz $d\mu(\lambda) \propto \lambda^{\gamma-1}\exp\left(-\frac{\lambda_a}{\lambda}-\frac{\lambda}{\lambda_b}\right)d\lambda$. The exponential factors cut off scaling behavior from below and above, and correspond to minimum and maximum abundances in the repertoire. This distribution, properly normalized, is the generalized inverse Gaussian [12]. For Poisson abundance models, the parameters λ_a and λ_b are strongly asymptotically correlated in the likelihood for fixed γ [13, 14]. This manifests as a ridge in parameter space that confounds likelihood maximization. A transformation that minimizes off-diagonal components of the Fisher information matrix is therefore introduced, resulting in the more orthogonal parameterization

$$d\mu_\theta(\lambda) = \frac{\xi^{-\gamma}}{2K_\gamma(\omega)}\lambda^{\gamma-1}e^{-\frac{\omega}{2}\left(\frac{\xi}{\lambda}+\frac{\lambda}{\xi}\right)}d\lambda, \qquad (4)$$

with parameter vector $\theta = (\gamma, \omega, \xi)$. $K_\gamma(\omega)$ denotes the modified Bessel function of the second kind, arising by imposing normalization. Excellent fits to naive and memory occupancy data were obtained with mixtures of two such distributions (see section IV). Lognormal and Pareto distributions were also considered, but produced substantially worse results.

Under the parameterization (4), the distribution (2) becomes

$$r_\theta(i) = \binom{L}{i}\frac{\xi^{-\gamma}}{2K_\gamma(\omega)}\int_0^\infty d\lambda\, \frac{\lambda^{\gamma-1}\left(e^{\frac{\lambda}{L}}-1\right)^i}{e^{\lambda+\frac{\omega}{2}\left(\frac{\xi}{\lambda}+\frac{\lambda}{\xi}\right)}}. \qquad (5)$$

Although not available in closed-form, these $L+1$ integrals can be approximated by quadrature to evaluate the

likelihood (3). Modeling as a mixture of two distributions of the form (4) adds a mixing parameter, $0 \leq \alpha \leq 1$, with $r_\theta(i) = (1-\alpha) r_{\theta_1}(i) + \alpha\, r_{\theta_2}(i)$.

C. Maximum likelihood diversity estimation

Direct maximization of the likelihood (3) is computationally formidable, as it constitutes a mixed integer nonlinear programming problem. However, it may be factorized in the suggestive form

$$\mathcal{L}(\theta, S|o) = \mathcal{L}_b(\theta, S|o)\, \mathcal{L}_m(\theta|o),$$

where we define the binomial

$$\mathcal{L}_b(\theta, S|o) = \binom{S}{s} r_\theta(0)^{S-s} \left(1 - r_\theta(0)\right)^s,$$

and zero-truncated multinomial

$$\mathcal{L}_m(\theta|o) = s! \prod_{i=1}^{L} \frac{1}{o_i!} \left(\frac{r_\theta(i)}{1 - r_\theta(0)} \right)^{o_i}.$$

An approach to approximate maximization of $\mathcal{L}(\theta, S|o)$, proposed by Sanathanan as conditional maximum likelihood estimation [15], is to first compute

$$\hat{\theta} = \arg\max_{\theta} \mathcal{L}_m(\theta|o),$$

which is independent of S and can be obtained by nonlinear numerical maximization of the log-likelihood $\ell_m(\theta|o) = \log \mathcal{L}_m(\theta|o)$. A constrained gradient ascent algorithm [16] was used in the present work. Differentiation gives gradient components of the form

$$\frac{\partial \ell_m(\theta|o)}{\partial \theta_j} = \frac{s}{1 - r_\theta(0)} \frac{\partial r_\theta(0)}{\partial \theta_j} + \sum_{i=1}^{L} \frac{o_i}{r_\theta(i)} \frac{\partial r_\theta(i)}{\partial \theta_j},$$

with

$$\frac{\partial r_\theta(i)}{\partial \theta_j} = \binom{L}{i} \int_0^\infty d\left(\frac{\partial \mu_\theta(\lambda)}{\partial \theta_j} \right) e^{-\lambda} \left(e^{\frac{\lambda}{L}} - 1 \right)^i,$$

which may be evaluated by quadrature for the parameterization (4).

Having computed $\hat{\theta}$, it remains to maximize the richness piece of the likelihood. A lemma due to Chapman [17] can be invoked to give

$$\begin{aligned} \hat{S} &= \arg\max_{S \in \mathbb{N}} \mathcal{L}\left(\hat{\theta}, S \middle| o\right) \\ &= \arg\max_{S \in \mathbb{N}} \mathcal{L}_b\left(\hat{\theta}, S \middle| o\right) \\ &= \left\lfloor \frac{s}{1 - r_{\hat{\theta}}(0)} \right\rfloor. \end{aligned}$$

Sanathanan's articulation of an asymptotic theory for the estimator $\hat{S}$ showed it to be equivalent to direct maximization of $\mathcal{L}(\theta, S|o)$ for large S. A corresponding approach was taken by Rodrigues [18] in an empirical Bayes treatment to approximate a posterior distribution for S. The density $\mu'_\theta(\lambda)$ is viewed as a prior which is realized in the repertoire for large S. Due to the large diversity of the BCR repertoire, we employ the diversity estimator $\hat{S}$, first investigating its accuracy via simulation.

D. Shannon diversity in a Poisson abundance model

To quantify the degree of uniformity in repertoire clonal abundance we derive a standard entropy-based index of diversity applied to a Poisson abundance model. For a repertoire with richness S and clone-wise population fractions given by $\pi_1, \pi_2, \ldots \pi_S$, the Shannon index [19] is defined as the information entropy of the clone-wise abundance distribution.

$$H = -\sum_{i=1}^{S} \pi_i \log \pi_i.$$

The maximum entropy, $H_o = \log S$, occurs when $\pi_i = 1/S$ for all clones. We define clonality, C, as the complement of the normalized Shannon entropy $C = 1 - H/H_o = 1 - H/\log S$. C ranges on the unit interval, with zero denoting maximally uniform abundance across clones and unity denoting the most disparity (dominated by a single clone).

In a Poisson abundance model, each clone, i, is assigned a Poisson frequency, λ_i, which is related to its population fraction, π_i, by $\lambda_i = \langle n \rangle \pi_i$, where $\langle n \rangle$ denotes the expected sample size.

$$\langle n \rangle = \sum_{i=1}^{S} \lambda_i.$$

With the measure parameterized by θ this becomes

$$\begin{aligned} \langle n \rangle_{S,\theta} &= S \int_0^\infty d\mu_\theta(\lambda) \lambda \\ &= S I_1(\theta), \end{aligned}$$

where we've defined the integral

$$I_1(\theta) = \int_0^\infty d\mu_\theta(\lambda) \lambda.$$

The Shannon index for a Poisson abundance model is then

$$\begin{aligned} H(S, \theta) &= -S \int_0^\infty d\mu_\theta(\lambda) \frac{\lambda}{\langle n \rangle} \log \frac{\lambda}{\langle n \rangle} \\ &= \log \langle n \rangle - \frac{S}{\langle n \rangle} \int_0^\infty d\mu_\theta(\lambda) \lambda \log \lambda \\ &= \log S + \log I_1(\theta) - \frac{I_2(\theta)}{I_1(\theta)}, \end{aligned}$$

with

$$I_2(\theta) = \int_0^\infty d\mu_\theta(\lambda) \lambda \log \lambda.$$

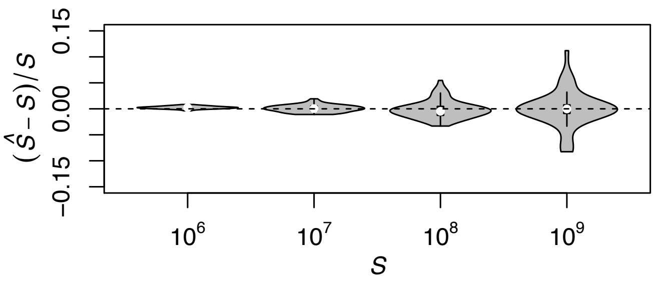

FIG. S1: **Performance of diversity estimation on simulated data.** One hundred simulations were performed for each of four diversity values, and Diversity estimates were computed for each. The resulting fractional errors are summarized as violin plots.

The clonality is then evaluated at the MLE as

$$C(\hat{S},\hat{\theta}) = 1 - \frac{H(\hat{S},\hat{\theta})}{\log \hat{S}} = \frac{1}{\log \hat{S}}\left(\log I_1(\hat{\theta}) - \frac{I_2(\hat{\theta})}{I_1(\hat{\theta})}\right).$$

For parameterization (4) the necessary integrals may be evaluated in terms of modified Bessel functions as

$$I_1(\hat{\theta}) = \xi \frac{K_{-\gamma-1}(\omega)}{K_\gamma(\omega)}$$

and

$$I_2(\hat{\theta}) = \frac{\xi}{K_\gamma(\omega)}\left(\log \xi \; K_{\gamma+1}(\omega) - \left[\frac{d}{dx}K_x(\omega)\right]_{x=-\gamma-1}\right)$$

It is trivial to extended this to a model with two mixed generalized inverse Gaussians.

IV. RESULTS

A. Simulation validation

To validate our methodology for inferring richness, simulations were performed by generating random draws from the likelihood (2). Fig. S1 shows violin plots for fractional error in diversity estimation for four sets of 100 simulations. Each violin is for a set of 100 simulations with identical diversity (S) and shows the distribution of the fractional error of the MLE, $(\hat{S} - S)/S$.

The values of S for the four sets are 10^6, 10^7, 10^8, and 10^9. For all four sets, the expected sample size is fixed at about 4.7 million cells. This is achieved by tuning the scale parameter ξ inversely as S. This is necessary to address a property of the sampling model: the expected sample size is proportional to both S and ξ, but we want expected sample size to be the same in all simulations as we tune S. Remaining parameters were fixed at $\gamma = -1$ and $\omega = 0.01$ across all simulations (values similar to those arising in analyzing real data). Even at the high end of diversity, the expected error is only a few percent, demonstrating the efficacy of conditional maximum likelihood estimation in estimating an unknown population parameter.

B. B cell diversity estimation

Details of diversity estimation applied to experimental data are presented in Fig. S2 and Table. S1, and diversity metric inferences are summarized in main text Fig. 2. Occupancy data with visualized fits of the MLE are shown in Fig. S2. Excellent fits are obtained for all data sets, as assessed by comparison to expectation values $\langle o_i \rangle = \hat{S}\; r_{\hat{\theta}}(i)$, $i = 1, 2, \ldots, L$, and with variation characterized by the quantile functions of the binomial marginal of likelihood (3) at $\hat{S}$ and $\hat{\theta}$. Estimated richness, clonality, and parameterization values are very consistent between the two samples of subject 1's naive BCR repertoire, and take on characteristic values according to cell population.

V. DISCUSSION

By synthesizing flow cytometry and replicate immunosequencing, approximate digital cell counting of memory and naive B cell repertoires of three adults was enabled, providing the deepest and most quantitatively robust characterization of the repertoire yet available. Diversity of the repertoire was inferred using a novel likelihood model devised for replicate-based presence-absence data. Estimates of both clonal richness and evenness of abundance distributions were attained, showing consistency across individuals, but distinct clustering by cell population. Across naive samples, the estimated richness is similar to the expected number of total naive B cells in circulation, suggesting that the typical naive B cell undergoes no proliferation prior to antigen stimulation. Memory richness is consistent with several divisions on average, but higher disparity in abundance (indicated by lower clonality), likely corresponding to clonal expansions in response to antigen stimulation.

[1] J. Bunge and M. Fitzpatrick, J. Am. Statist. Assoc. **88**, 364 (1993).

[2] C. Vollmers, R. V. Sit, J. A. Weinstein, C. L. Dekker, and

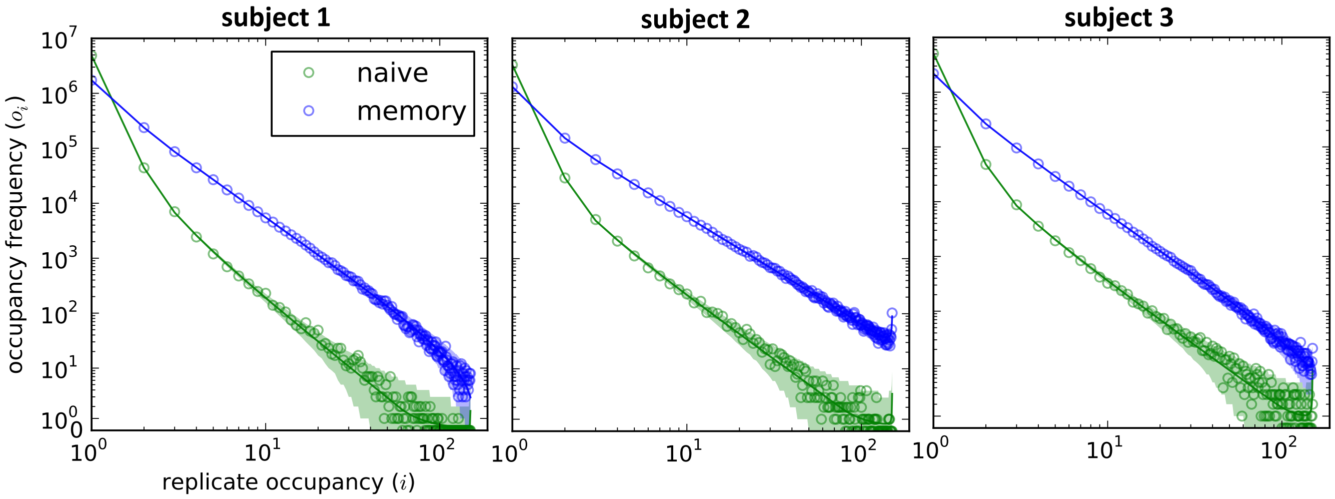

FIG. S2: **Diversity estimation results.** Replicate occupancy data (circles) for naive (green) and memory (blue) samples, with expected occupancies at the MLE (solid lines) and 99% marginal intervals (colored bands), indicating goodness of fit. Data for the second naive sample for patient 1 is omitted because results were not visually distinct. See Table S1 for numerical details of MLE results.

TABLE S1: **MLE details.** Diversity, ($\hat{S}$), parameterization, $\hat{\theta} = \theta_1 + \alpha\theta_2$, and clonality, $C(\hat{S}, \hat{\theta})$, for all samples.

subject	population	$\hat{S}$ (10^9)	θ_1	θ_2	α	$C(\hat{S}, \hat{\theta})$
**1**	**naive 1**	2.39	(-1.46, 9.04 10^{-4}, 8.69)	(-.097, .379 , 9.83 10^{-4})	.944	.033
	**naive 2**	2.46	(-1.47, 9.44 10^{-4}, 8.69)	(-.102, .410, 8.74 10^{-4})	.945	.031
	**memory**	.0527	(-1.13, 7.98 10^{-2}, 9.07)	(-.0722, .293, .0132)	.960	.043
**2**	**naive**	1.11	(-1.23, 7.28 10^{-3}, 8.69)	(-.0944, .416, 1.68 10^{-3})	.998	.029
	**memory**	.0765	(-.860, 3.67 10^{-2}, 9.08)	(-.0706, .285, 6.38 10^{-3})	.973	.042
**3**	**naive**	1.97	(-1.25, 1.80 10^{-3}, 8.69)	(-.0962, .393, 1.39 10^{-3})	.989	.030
	**memory**	.116	(-1.13, 5.03 10^{-2}, 9.07)	(-.0778, .287, 6.67 10^{-3})	.968	.042

S. R. Quake, Proc. Natl. Acad. Sci. U.S.A. **110**, 13463 (2013).

[3] H. S. Robins, P. V. Campregher, S. K. Srivastava, A. Wacher, C. J. Turtle, O. Kahsai, S. R. Riddell, E. H. Warren, and C. S. Carlson, Blood **114**, 4099 (2009).

[4] I. J. Good and G. H. Toulmin, Biometrika **43**, 45 (1956).

[5] B. Efron and R. Thisted, Biometrika **63**, 435 (1976).

[6] T. Daley and A. D. Smith, Nat. Meth. (2013).

[7] G. A. Rempala, M. Seweryn, and L. Ignatowicz, J. Theor. Biol. **269**, 1 (2011).

[8] J. Greene, M. R. Birtwistle, L. Ignatowicz, and G. A. Rempala, J. Theor. Biol. **326**, 1 (2013).

[9] C. S. Carlson, R. O. Emerson, A. M. Sherwood, C. Desmarais, M.-W. Chung, J. M. Parsons, M. S. Steen, M. A. LaMadrid-Herrmannsfeldt, D. W. Williamson, R. J. Livingston, et al., Nat. Comms. **4** (2013).

[10] H. S. Wilf, *Generatingfunctionology* (Academic Press, London, 2000).

[11] T. Mora, A. M. Walczak, W. Bialek, and C. G. Callan, Jr, Proc. Natl. Acad. Sci. U.S.A. **107**, 5405 (2010).

[12] B. Jørgensen, *Statistical Properties of the Generalized Inverse Gaussian Distribution*, vol. 9 of *Lecture Notes in Statistics* (Springer, New York, NY, 1982).

[13] G. Z. Stein, W. Zucchini, and J. M. Juritz, J. Am. Statist. Assoc. **82**, 938 (1987).

[14] G. E. Willmot, J. Am. Statist. Assoc. **83**, 517 (1988).

[15] L. Sanathanan, J. Am. Statist. Assoc. **72**, 669 (1977).

[16] C. Zhu, R. H. Byrd, P. Lu, and J. Nocedal, ACM Trans. Math. Softw. **23**, 550 (1997).

[17] D. Chapman, *Some properties of the hypergeometric distribution with applications to zoological sample censuses*, University of California publications in statistics (University of California Press, 1951).

[18] J. Rodrigues, L. A. Milan, and J. G. Leite, Biom. J. **43**, 737 (2001).

[19] C. Shannon, Bell Syst. Tech. J. **27**, 379 (1948).
